# Supplementary material for: Comparing methods for deriving the auditory brainstem response to continuous speech in human listeners
Source: Imaging Neurosci (Camb). 2025 Jun 3;3:IMAG.a.19. doi: 10.1162/IMAG.a.19 (PMC12319856; doi:10.1162/IMAG.a.19)
Supplement: Supplementary Material [file imag.a.19_supp.pdf]

Supplemental Materials

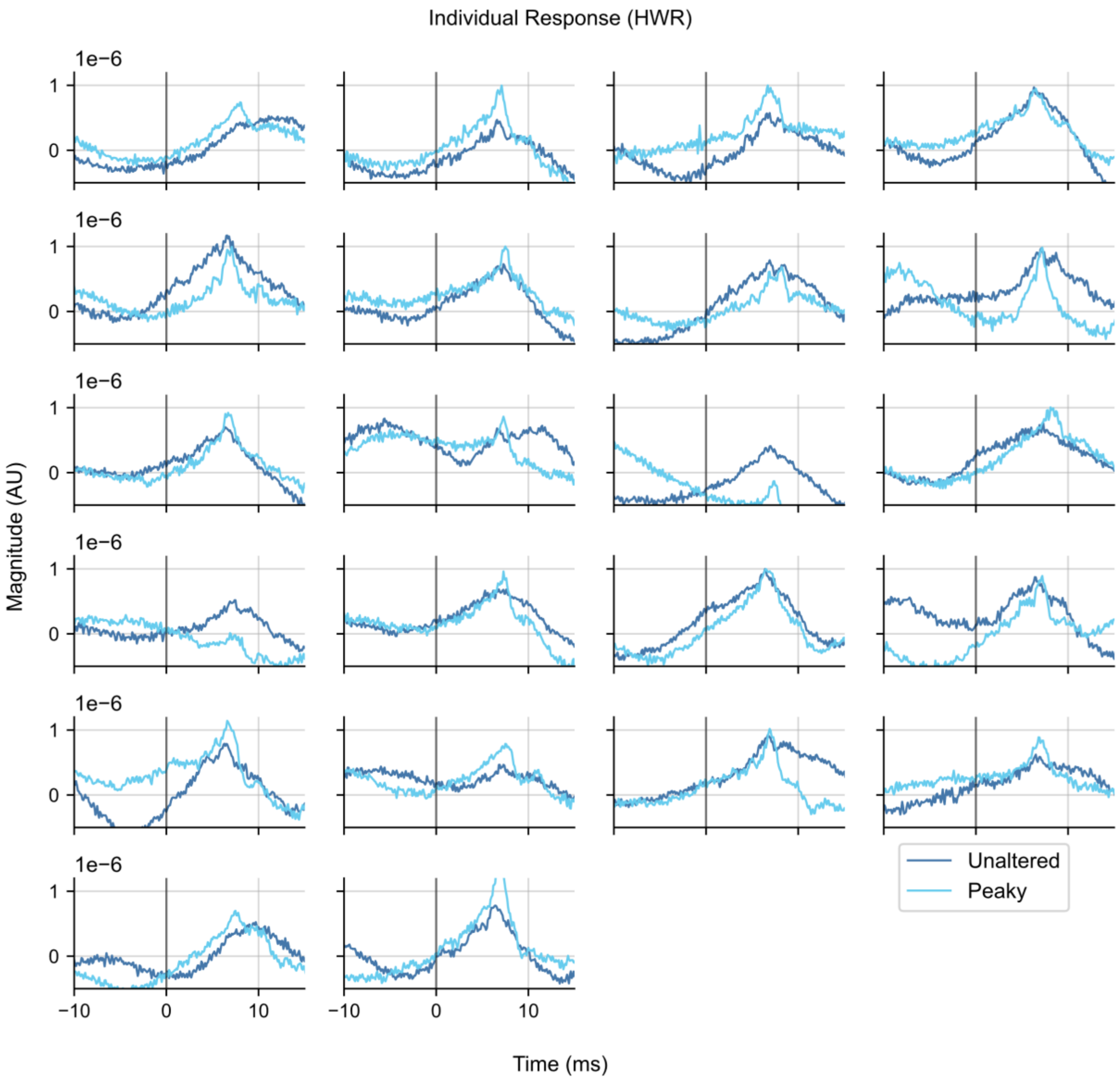

Figure S1. Individual ABR waveforms for unaltered and peaky speech derived from HWR regressor.

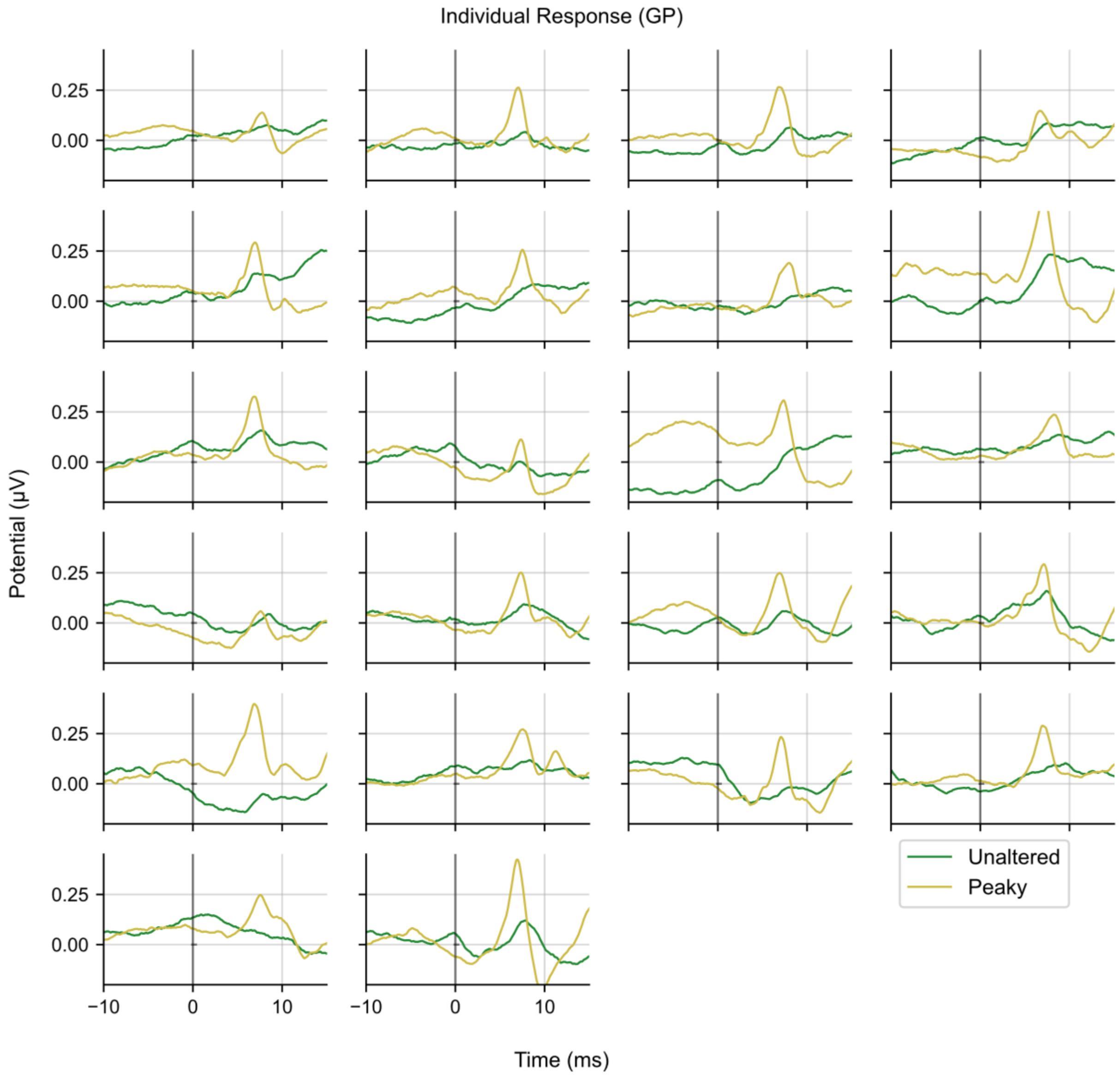

Figure S2. Individual ABR waveforms for unaltered and peaky speech derived from GP regressor.

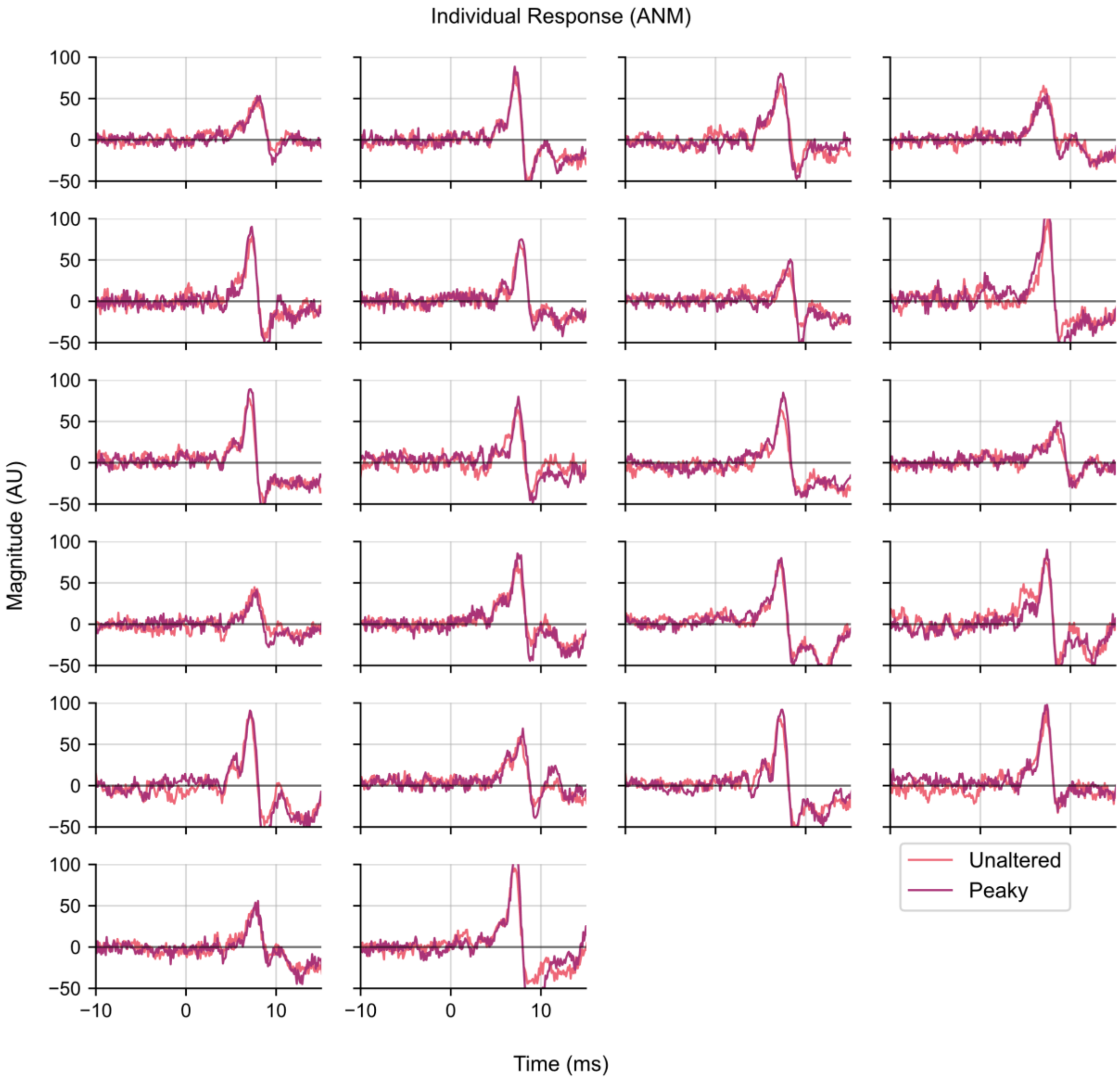

Figure S3. Individual ABR waveforms for unaltered and peaky speech derived from ANM regressor.

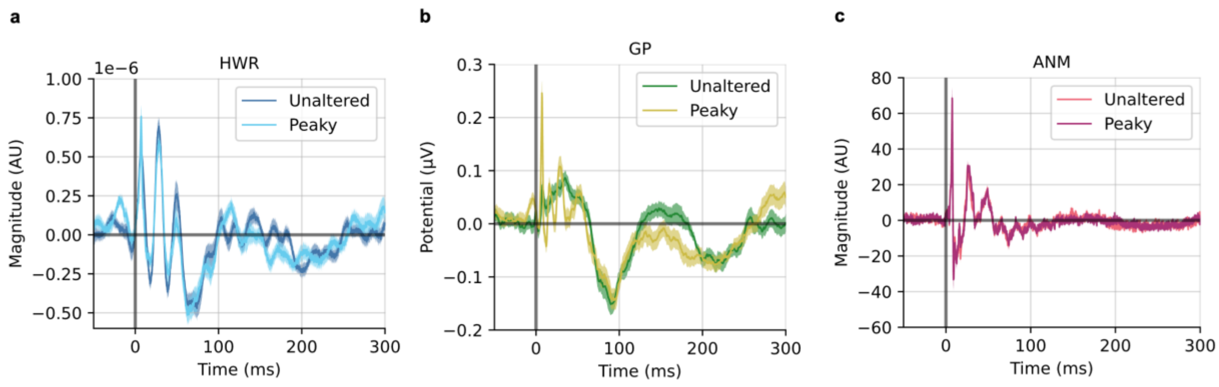

Figure S4. Grand averaged waveforms in time window of [-50, 300] ms for unaltered and peaky speech derived from HWR (**a**), GP (**b**) and ANM (**c**) regressor. Shaded area show ± 1 SEM (n = 22).

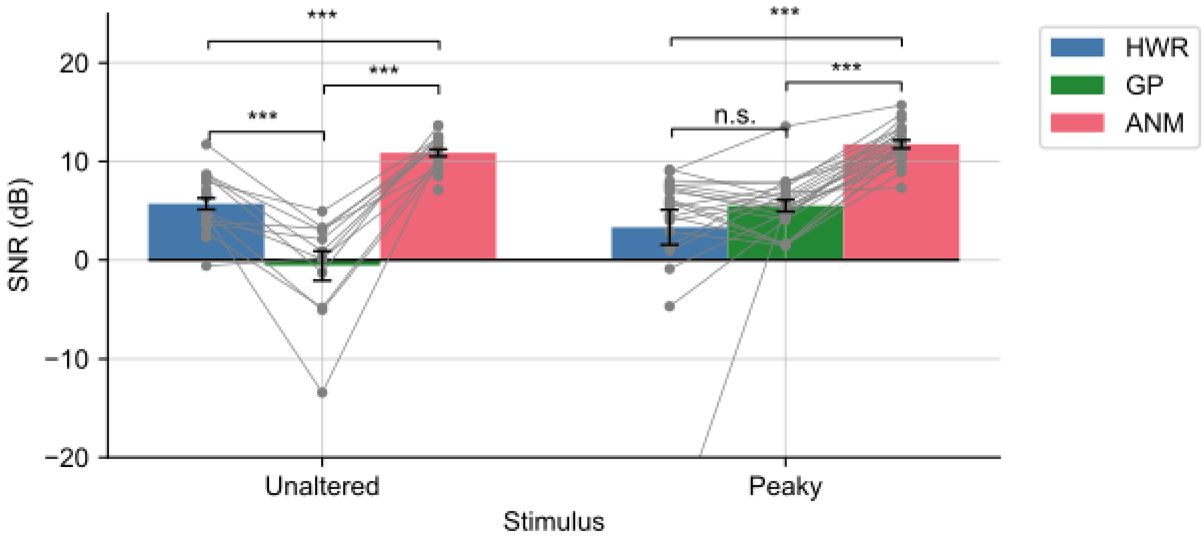

Figure S5. SNR analysis for the derived ABRs using analysis time window of [0, 30] ms. The bar represents the averaged SNR across subjects and the grey dots with lines are the SNRs for each individual subject.

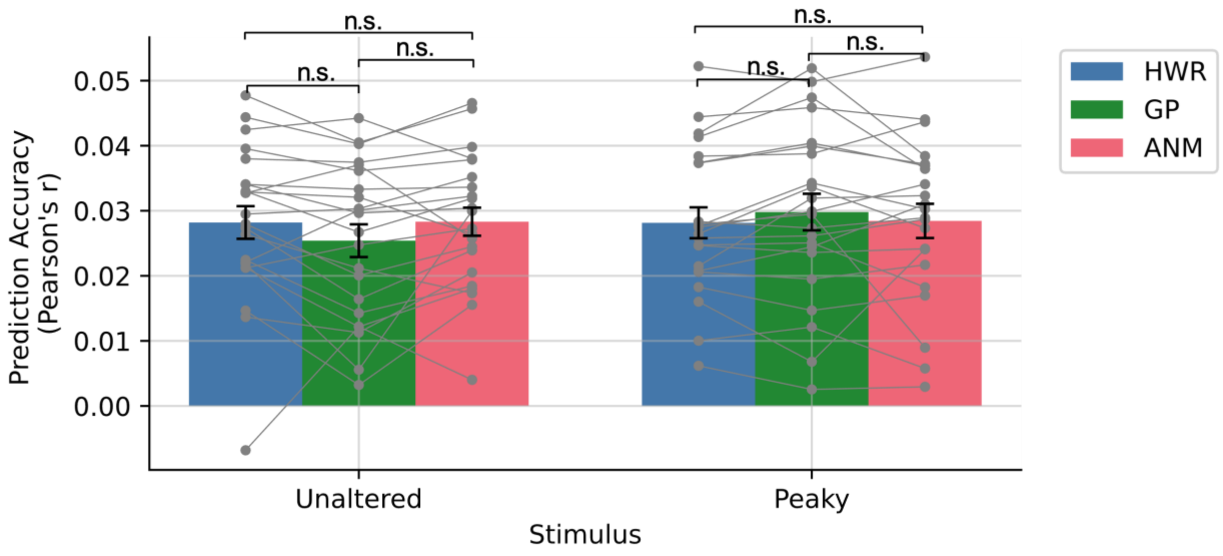

Figure S6. Prediction accuracy as in broadband correlation coefficient with full kernel (0–200 ms).

Table S1. The Mixed Model for SNR analysis using [0, 15] ms time window.

	**Coefficient**	**Z**	**P**
**Intercept (HWR unaltered)**	3.838	4.433	<0.001
**Regressor (GP)**	-5.184	-4.201	<0.001
**Regressor (ANM)**	8.450	7.483	<0.001
**Stimulus (Peaky)**	1.477	1.308	0.191
**Regressor (GP)*stimulus (Peaky)**	9.121	5.486	<0.001
**Regressor (ANM)*stimulus (Peaky)**	-0.594	-0.375	0.708

Table S2. The Mixed Model for Prediction accuracy as the correlation coefficient (Pearson's r).

	**Coefficient**	**Z**	**P**
**Intercept (HWR unaltered)**	0.004	2.590	0.010
**Regressor (GP)**	0.001	0.722	0.471
**Regressor (ANM)**	0.004	2.100	0.036
**Stimulus (Peaky)**	0.004	1.966	0.049
**Regressor (GP)*stimulus (Peaky)**	0.002	0.764	0.445
**Regressor (ANM)*stimulus (Peaky)**	0.000	0.157	0.875
